# Cholera’s global resurgence: urgent appeal for enhanced vaccine availability in Nigeria, and beyond

**DOI:** 10.1177/20499361241303431

**Published:** 2024-12-03

**Authors:** Roland I. Stephen, Jamiu S. Olumoh, Jimmy A. Reyes, Malachy I. Okeke, Jennifer A. Tyndall, Nura Umaru, Oyelola A. Adegboye

**Affiliations:** School of Doctoral Studies, Unicaf University Cyprus, Larnaca, Cyprus; Department of Public Health, Modibbo Adama University Teaching Hospital, Yola, Adamawa State, Nigeria; Department of Mathematics and Statistics, American University of Nigeria, Yola, Adamawa State, Nigeria; Department of Nursing and Public Health, University of Northern Iowa, Cedar Falls, Iowa, USA; Department of Natural and Environmental Sciences, American University of Nigeria, Yola, Adamawa State, Nigeria; Department of Natural and Environmental Sciences, American University of Nigeria, Yola, Adamawa State, Nigeria; Department of Medicine, Federal Teaching Hospital, Gombe, Gombe State, Nigeria; Menzies School of Public Health, Charles Darwin University, Casuarina, NT 0810, Australia

**Keywords:** epidemiology, infectious disease, oral cholera vaccine, public health, vibrio cholerae, water sanitation and hygiene

The recent cholera outbreak in Lagos, Nigeria’s commercial nerve centre, calls for urgent and concerted action to prevent further spread. While Nigeria is endemic to cholera, Lagos has historically been spared.^1^ However, its status as a pivotal commercial hub in Africa and globally places it at significant risk of amplifying the spread of cholera worldwide.

In light of this resurgence, declared a ‘global resurgence of cholera’ by the World Health Organization (WHO) on 21 June 2024, urgent measures are imperative. As of 25 June 2024, Lagos has reported 43 confirmed cases and 29 deaths, with 579 suspected cases.^2^ According to the Nigerian Centre for Disease Control and Prevention (NCDC), the country has recorded 1528 suspected cases and 53 deaths this year as of 24 June 2024.^3^ This surge with a case fatality rate (CFR) of 5.0% and 3.5% in Lagos and nationally is far higher than the WHO recommended CFR benchmark of 1.0%. This underscores the need for swift intervention to prevent further escalation of the disease outbreak. Furthermore, the WHO’s classification of this surge as a grade 3 emergency highlights its severity across multiple WHO regions, with 195,000 cases and 1900 deaths recorded globally in 24 countries in 2024.^4^ This calls for a robust global response, a call to action that extends beyond traditional water, sanitation and hygiene measures, and requires the collective effort of all involved in infectious disease control and global health.

Over the past five decades, Nigeria has experienced several major cholera outbreaks, notably in 1991, 1999, 2010, 2019 and 2024, respectively.^5^ The first recorded outbreak in the country occurred in 1972 when Lagos State documented 22,931 cases and 2945 deaths.^6,7^ The 1991 outbreak recorded 59,478 cases and 7654 deaths (CFR: 12.9%), while in 2010, there were 41,787 cases and 1716 deaths (CFR: 4.1%), affecting over two-thirds of the states.^5^ Subsequent outbreaks in 2014 and 2018 recorded 4996 cases (CFR: 2%) and 43,996 cases with 836 deaths, respectively, impacting a similar proportion of states.^5^ Data from the Nigeria Centre for Disease Control (NCDC; Figure 1 top panel) showed that cholera was highly concentrated in the northeastern part, particularly in Borno state, with incidence rates up to 160 cases per 100,000 people in 2022. By 2023, cholera cases have lowered (up to 16 per 100,000), however, it has spread to more states in the country. In 2024, cholera cases seem to be further reduced and localised. This trend suggests a possible improvement in cholera control measures over time, highlighting the need for targeted interventions in states that continue to experience outbreaks.

**Figure 1.**
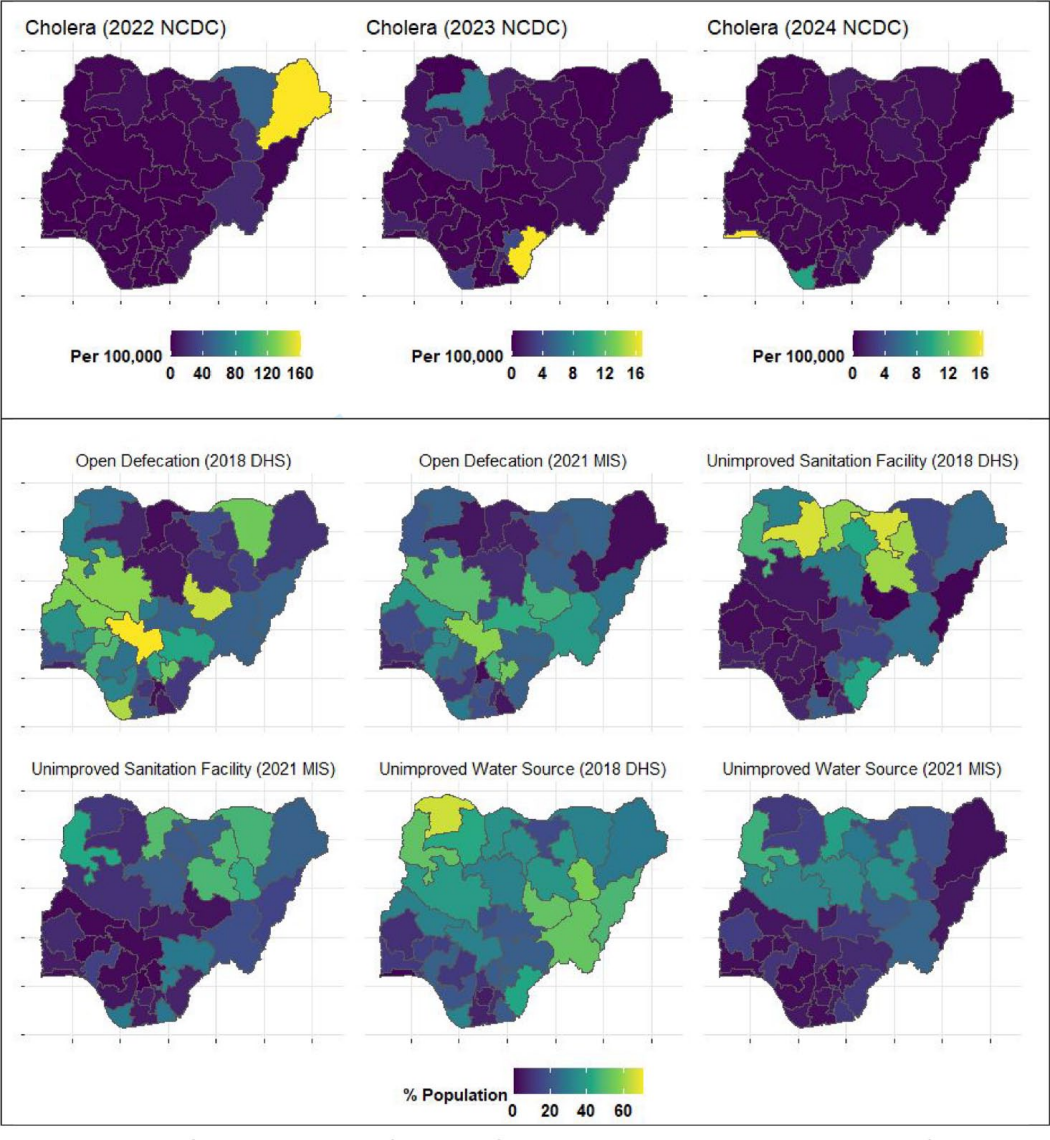
Maps of Nigeria showing cholera incidence rates per 100,000 (top panel), and the percentage of the population using open defecation, unimproved sanitation facilities and unimproved water sources (middle and bottom panel). Cholera incidence data were sourced from the NCDC, while sanitation and water access estimates were derived from the 2018 Nigerian DHS and the 2021 MIC survey. Unimproved water sources include unprotected wells, unprotected springs and surface water (e.g., river, dam, lake, pond, stream, canal, irrigation channel). Unimproved sanitation facilities comprise flush-to-somewhere else systems, pit latrines without slabs, bucket toilets, hanging toilets, shared facilities and open defecation. DHS, demographic and health surveys; MIC, multiple indicator clusters; NCDC, Nigeria Centre for Disease Control.

Health authorities have implemented various policies to address the recurrent cholera epidemics, including improving Water, Sanitation and Hygiene. Although these measures have had some success, outbreaks continue to occur annually.^5^ To further improve WASH services, the Nigerian government launched a 13-year National Action Plan, alongside adopting advanced water purification technologies such as ozone therapy, electrocoagulation and nanotechnology.^5^ Based on available data from the Nigerian Demographic and Health Surveys (DHS) and the Multiple Indicator Clusters survey, significant reductions were observed in the percentage of the population using open defecation, unimproved sanitation facilities and unimproved water sources across Nigerian states for the years 2018 and 2021 (Figure 1).

The ‘Global strategy on cholera control, ending cholera: a global roadmap to 2030’, established in 2017, aims to reduce cholera deaths by 90% by the end of the decade.^8^ Central to achieving this goal is scaling up the availability of oral cholera vaccine (OCV), particularly in developing nations where the supply remains inadequate. Given the mounting evidence linking climate change to the increased frequency of cholera outbreaks,^9^ strategic planning for OCV distribution is crucial. Over the years, the world has witnessed several cycles of cholera outbreaks, underscoring the persistent threat posed by this acute gastroenteritis.^9^ Cholera, caused by a bacterium, *Vibrio cholerae*, is an acute diarrheal illness that can lead to severe dehydration and death if left untreated.^10^ It primarily affects populations with inadequate access to safe water and basic sanitation(Figure 2).^10^ The transmission of cholera through contaminated water and food highlights the critical role of vaccination and improved sanitation in its prevention. Despite significant advancements in understanding and managing cholera, it remains a serious public health threat, particularly in developing countries.

**Figure 2.**
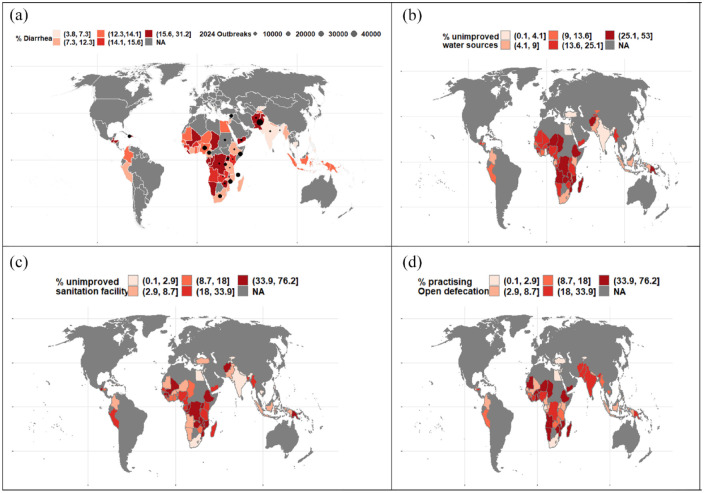
Map illustrating the global (a) prevalence of diarrhoea among children under five, based on the percentage of under-five children who experienced diarrhoea in the 5 years preceding the survey [The dots represent the current global cholera outbreaks (January–May 2024) reported by WHO (4)], percentage of the population living in households, (b) without improved water sources on the premises, (c) with an unimproved sanitation facility and (d) practising open defecation (no facility/bush/field). All estimates ((b)–(d)) are based on the most recent DHS/AIS/MIS data set after 2010. Unimproved water sources are unprotected wells, unprotected springs and surface water (river/dam/lake/pond/stream/canal/irrigation channel). Unimproved sanitation facilities include flush-to-somewhere else, pit latrines without slabs, bucket toilets, hanging toilets, shared facilities and open defecation. AIS, AIDS indicator surveys

In conclusion, addressing the cholera resurgence requires a coordinated global effort to enhance vaccine availability and implement comprehensive public health measures. Failure to act decisively risks further outbreaks with far-reaching consequences for global health and economic stability. Given the multi-faceted challenges faced in tackling Cholera in Nigeria, particularly in areas with limited assess due to security concerns, poor sanitation and insufficient healthcare infrastructure, it is critical to adopt a multi-faceted approach to control and eventually eradicate the disease. Recommendations include intensifying efforts to improve water, sanitation and hygiene (WASH) infrastructure in rural and urban settings. This involves increasing funding for WASH, ensuring the effective use of rapid diagnostic tests and increasing their availability in affected communities.

## Declarations

**Ethics approval and consent to participate:** Not applicable. All data sets used in the analyses are publicly available at https://www.dhsprogram.com.

**Consent for publication:** Not applicable.

**Availability of data and materials:** All datasets presented in this study are publicly applicable at https://www.dhsprogram.com and https://ncdc.gov.ng/
